# Anticipating the impact of the COVID-19 pandemic on TB patients and TB control programmes

**DOI:** 10.1186/s12941-020-00363-1

**Published:** 2020-05-23

**Authors:** Toyin Togun, Beate Kampmann, Neil Graham Stoker, Marc Lipman

**Affiliations:** 1 Vaccines and Immunity Theme, Medical Research Council Unit The Gambia at the London School of Hygiene & Tropical Medicine (MRCG at LSHTM), Atlantic Boulevard, Fajara, The Gambia; 2 Faculty of Infectious and Tropical Diseases, London School of Hygiene and Tropical Medicine (LSHTM), Keppel Street, London, UK; 3 UCL Centre for Clinical Microbiology, Royal Free Campus, University College London, London, UK; 4 UCL-TB & UCL Respiratory, Division of Medicine, University College London, London, UK; 5 Respiratory Medicine, Royal Free London NHS Foundation Trust, London, UK

**Keywords:** Tuberculosis, COVID-19, Pandemic, Poverty, Africa, Children, Isolation, Transmission, Global, Co-morbidity

## Abstract

The COVID-19 pandemic has currently overtaken every other health issue throughout the world. There are numerous ways in which this will impact existing public health issues. Here we reflect on the interactions between COVID-19 and tuberculosis (TB), which still ranks as the leading cause of death from a single infectious disease globally. There may be grave consequences for existing and undiagnosed TB patients globally, particularly in low and middle income countries (LMICs) where TB is endemic and health services poorly equipped. TB control programmes will be strained due to diversion of resources, and an inevitable loss of health system focus, such that some activities cannot or will not be prioritised. This is likely to lead to a reduction in quality of TB care and worse outcomes. Further, TB patients often have underlying co-morbidities and lung damage that may make them prone to more severe COVID-19. The symptoms of TB and COVID-19 can be similar, with for example cough and fever. Not only can this create diagnostic confusion, but it could worsen the stigmatization of TB patients especially in LMICs, given the fear of COVID-19. Children with TB are a vulnerable group especially likely to suffer as part of the “collateral damage”. There will be a confounding of symptoms and epidemiological data through co-infection, as happens already with TB–HIV, and this will require unpicking. Lessons for COVID-19 could be learned from the vast experience of running global TB control programmes, while the astonishingly rapid and relatively well co-ordinated response to COVID-19 demonstrates how existing programmes could be significantly improved.

## Background

Every year, March 24th is celebrated as Annual World TB Day. This is usually acknowledged across the world by a wide range of activities, to highlight the unsettling fact that *Mycobacterium tuberculosis*, a human-specific pathogen, is still a scourge after millennia of coexistence. It has recently reclaimed top spot as the infectious disease that kills more people in the world than any other infectious pathogen.

This year, however, its show was stolen by a newly-emerging pandemic caused by a novel pathogen, Severe Acute Respiratory Syndrome Coronavirus-2 (SARS-CoV-2), which has jumped from animals to humans and causes the disease named COVID-19—a flu-like disease characterised by severe acute respiratory distress in its most severe form. By the first week of April 2020, a million cases of COVID-19 disease had been diagnosed worldwide, including at least 50,000 deaths since the beginning of the epidemic in January 2020. World TB Day itself was eclipsed, with events cancelled throughout the world, including our own annual London symposium [1].

In an extraordinarily short time, COVID-19 has supplanted everything—normal activity, non-SARS-CoV-2-related discussion, research, clinical trials, etc. It can seem as if things that were previously hugely significant issues are no longer important.

So, what about TB when COVID-19 is so front-and-centre globally? Clearly TB has not disappeared, but does anyone still care? And if so, are we able to do more than watch as an existing global health problem becomes increasingly neglected—leading to predictable outcomes? On the plus side, with such a long history of intense research and infectious disease management, can TB teach the world anything about how we should deal with COVID-19?

Tuberculosis remains a global health emergency and needs our attention more than ever, given that significant resources are now being diverted to COVID-19 management. To lose sight of the unfinished business of tuberculosis control will jeopardise important milestones, gains and ambitions, and we believe that now more than ever is the time to care about TB in adults and children^1^. In this manuscript, we cover areas of particular concern for both epidemics facing us today. This reflection was developed from a video conversation held by the participants on 20 March 2020 and posted online following the cancellation of our World TB Day 2020 event [2] .

## Scope

We discussed issues of how COVID-19 would affect control programmes: prioritisation of services, availability of drugs, the effect on hard-to-reach and low-income communities and the role of stigmatisation, how paediatric TB disease might be affected, the possibility of increased disease transmission or disease susceptibility, and the problems caused by likely co-morbidity. We thought it important to look at the situations in both high-income countries (HICs) and low and middle-income countries (LMICs), and we used the UK and Africa as exemplars with which we are most familiar. We also reviewed how control programmes for each disease could learn from each other.

We are of course not the only people reviewing and comparing these two pandemics, and we acknowledge the contributions by Wingfield et al. [3] who discussed the negative effect of COVID-19 on TB patients and control programmes, and The Union supported by TB Alert [4, 5] who actively compared and contrast the two diseases, and focused on ways in which TB expertise can be useful for COVID-19 control.

## The situation in the UK and Africa

### The UK

As an example of an HIC, the UK has a significant TB problem with approximately 4500 new cases a year, particularly in London [6]. There is a specific integrated TB control programme with allocated staff managing all aspects of diagnosis, treatment, contact tracing, outbreak investigation, latent disease, dialogue with communities, and linking to other relevant charities and organizations. In recent years, the numbers of cases have fallen, and multiple and extensively drug-resistant cases have been successfully treated. This TB control programme lies within a single National Health Service, administered through separate trusts, with healthcare free at the point of care for all citizens.

At the time of our video discussion, the UK was in the middle of the first wave of COVID-19, with the population under lockdown, number of cases and deaths still rising—putting a tremendous strain on the NHS as it struggled to cope with this, and much of normal clinical practice put on hold. London was the UK’s epicentre. Governmental loans to businesses and freelance workers, and paid sick leave in a high proportion of cases, was put in place to minimise short-term economic damage. Working from home was encouraged for many through a good internet structure.

### Africa

Africa has a much greater TB burden. In 2018, Africa had an estimated 24% of the 10 million global incident cases of TB and the region also has the smallest rate of decline of TB globally [7]. Although every country within Africa will have a different health system, they are limited and quality of TB care is sub-optimal. A 13-country patient-pathways analysis that included six African countries with the highest TB burden on the continent found that less than 50% of estimated TB patients had documented treatment success [8]. Also, a cascade of TB patient care analysis in South Africa reported that only 53% of TB patients in South Africa were diagnosed and successfully treated [9].

By 1st April, the COVID-19 pandemic had started in Africa, with 3766 cases and 95 deaths [10]. A large rise in cases of COVID-19 was expected as countries ramped up their testing and diagnostic capacity for COVID-19 [11] in the context of already stretched and poorly-resourced health care systems.

## How will COVID-19 impact on TB control in the UK?

It’s already apparent that there will be a need to prioritise the care that TB services can deliver. TB control will focus on TB disease rather than prevention (such as the management of latent, non-transmissible TB infection). This arises in part because of a TB workforce being re-directed to manage COVID-19, others being off sick or self-isolating (up to 30% of healthcare staff); and the concern that too much social contact within a clinic is likely to result in more viral transmission. The need to reduce contact with patients who see their TB service as a source of both social and medical support may result in reduced adherence and worse outcomes. This will affect those who are least able to self-care—in other words the very people who most need TB services.

A potential unintended consequence of the UK and many other Government’s promotion and enforcement of isolation is that socially-disadvantaged and homeless populations may be more likely to come together in hostels or other settings and hence increase their risk of acquiring and passing on infection. They are also perhaps less able to protect themselves from contact transmission by regular hand washing and use of sanitizers. In households who are self-isolating, a coughing adult may have a viral infection, though could have TB that is then inadvertently passed on to other family members.

Pre-COVID-19 and Brexit, there were regular anti-TB therapy stock-outs. This is likely to get worse as supply chains become harder to sustain during a time of prolonged infection, illness and economic shut down. As a result, not only will TB services have to modify treatment regimens in line with what drugs are available, but there will also be errors in prescribing—leading to more adverse effects and treatment failures. These are issues that every high income country affected by COVID-19 faces.

## How is COVID-19 going to impact on TB control in Africa?

One of the cardinal objectives of the WHO’s End TB Strategy [12], which proposes the target of ending the TB epidemic in the next 15 years, is the provision of high-quality and patient-centred care for TB patients based on the their human rights [13]. This very ambitious target requires sustained political and financial commitment from African countries toward the provision of basic essential universal health care, and specifically for TB diagnostic, prevention and treatment services. However, the studies cited earlier suggest that, even prior to the onset of the COVID-19 pandemic, the quality of TB care in Africa is sub-optimal. COVID-19 will potentially worsen patient care and TB control efforts in Africa, given the likely negative impact of the pandemic at the macroeconomic, health system and individual levels in Africa.

COVID-19 is expected to result in a global economic recession in 2020 with the economic downturn particularly worse for the emerging markets and low-income countries, as recently communicated by the International Monetary Fund [14]. Without gainsaying, the impact of the global economic recession will be worse on the LMICs of Africa—many of which were either in borderline or full economic recession prior to the coronavirus pandemic.

While HICs are now releasing economic stimulus packages, this will be practically impossible in African LMICs, who often depend on donor funds and natural resources from extractive industries. As these countries are now setting up and/or ramping-up their COVID-19 responses in the midst of a global economic downturn, we have already seen the diversion of political will, scarce financial and the limited human resources in the hitherto weak health system to the COVID-19 responses. As an example, Nigeria that has the largest burden of TB in Africa and is one of the 30 high TB burden countries globally recently announced that 300 GeneXpert machines in the country will be diverted to efforts to scale-up COVID-19 diagnosis [15]. This will definitely have a huge negative impact on basic and essential TB control efforts, including routine diagnosis of TB cases, treatment monitoring by direct observation, provision of TB preventive therapy, and contact tracing among others.

Tuberculosis in Africa is a disease of poverty as in all LMICs, most often afflicting the poorest people in the society who also have the least access to basic health care. We also know that socio-economic circumstances are a crucial determinant of health outcomes, in particular for people with tuberculosis. Many of the socioeconomic and behavioural factors that could enhance transmission of coronavirus in Africa are also the recognised factors that enhance transmission of *M. tuberculosis*. While patients with chronic cough might hesitate to seek care due to the ‘new’ stigma and fear associated with COVID-19, they are very likely going to meet an unprepared and overwhelmed health system when, or if, they do. On the other hand, the interventions proposed to “flatten the curve” in COVID-19 responses e.g. physical distancing, regular handwashing, whole community lockdowns, etc. are going to be disproportionately more challenging for the poorest who most often live in overcrowded conditions, with no or limited access to drinking water, and earn meagre incomes from daily wages. Furthermore, very little is known about the pathobiological mechanisms of the COVID-19 caused by the novel SARS-CoV-2 especially in populations in Africa with a relatively higher prevalence of HIV infection, TB, anaemia and malnutrition, as well as in patients with significant post-TB lung damage.

Taken together, it is clear that the impact of COVID-19 in Africa is going to be disproportionately worse on the very poor and the least-advantaged people, who already bear the largest burden of both communicable and non-communicable diseases, and also have the least access to basic healthcare. These will include many undiagnosed and current TB patients who are, as a result, very likely to have worse outcomes from their TB.

## The likely impact of COVID-19 on children with TB

Amongst the 1.5 million annual deaths from TB, an estimated 205,000 occur in children with the majority occurring in resource-poor settings [7]. Child survival from TB depends on timely diagnosis, prompt initiation of treatment, community and health systems support for continuous availability of child friendly medication as well as prevention of transmission from sputum-smear positive index cases-usually adults-to vulnerable young children in households.

These important elements of the cascade of care are at stake at a time when resources will be focused on providing care for individuals affected by COVID-19. The diagnosis of childhood TB is not only made at dedicated clinics of National TB control programs but equally in generic child health clinics and hospital wards. Many of these facilities will be closed or overwhelmed with other tasks, and as a result diagnostic opportunity in children will be missed. The majority of children in LMIC are not seen by dedicated paediatric specialists, and many general physicians and nurses usually available for their care will be seconded to dealing with adult patients affected by severe respiratory symptoms of COVID-19 instead.

Available specialist expertise in respiratory medicine switched to diagnosing and treating this new viral disease, which does not appear generally to have similarly severe manifestations in children compared to adults. Hence it will be assumed that children will cope better and do not need similar attention from the health services during the COVID-19 pandemic. We are already witnessing this trend in HIC where routine clinics are cancelled and paediatric intensive care beds are handed over to adult services. Laboratories are likely to be overwhelmed with analyses of respiratory specimens sent for COVID-19 rather than *M. tuberculosis* or other pathogens, and once the eagerly anticipated Xpert cartridges for COVID-19 are rolled out it is easy to imagine that GeneXpert platforms will be seconded for COVID-19 diagnostics.

These issues are not specific for childhood TB as adult TB services are likely to be similarly affected. However, the timely diagnosis of TB in children is even more essential to prevent deaths. Families are reluctant to bring unwell children to the hospitals for investigation as everyone is discouraged from using health services at this stage, unless severely unwell. Apart from TB meningitis, TB rarely presents as an acute, severe illness in children but progresses silently until tipping points are reached. Such subtle presentations are likely to be missed if children cannot be reviewed regularly. Given the overlapping presentations of TB and pneumonia in children in the first place, many children are initially placed on a trial of antibiotics but ought to be reviewed in a timely fashion. This does not happen with severely-stretched health services.

Another important part of services for children is the provision of preventive therapy (IPT) for TB infection in the community, which requires resources to contact trace, screen and eventually implement drug therapy. These services are rudimentary in LMICs at the best of times. IPT works, and given that the under 5 year olds are particularly at risk of progressing to TB disease in its absence, the number of cases of childhood TB will most likely rise as a consequence. Whether such figures can even be captured when services are near to breaking-point remains to be seen.

As most TB in young children is acquired in their own household, social distancing measures that keep a family together for long periods of time are likely to result in more exposure of children to infectious TB index cases. Contact screening for COVID-19 should therefore include questions about TB in the household in order to protect young children from additional risks.

## Stigmatisation

Tuberculosis stigmatisation is a problem in many settings, though more so in LMICs. It is likely to rise and be confounded by COVID-19. Stigma is associated with fear, and fear of COVID-19 will increase. This has already been seen in the cases primarily imported into Africa—with stigmatization being directed not only at the affected patients but also their carers and family. This applies equally to healthcare workers who are likely to be managing both COVID-19 and TB.

The 2020 World TB Day had an emphasis on de-stigmatizing TB, but we should be de-stigmatizing any infectious disease. People do not go around spreading disease deliberately within their communities. As such, there is the need for stronger community engagement, including families and community-based groups being enabled to act as advocates.

The symptoms of TB and COVID-19 can be similar, with for example cough, fever, breathlessness and malaise being common in both. Not only can this create diagnostic confusion, but TB patients who are already stigmatized for coughing will be even more likely to be viewed with concern in LMICs, given the fear of COVID-19. This could result in people being afraid to present to healthcare services when they have such symptoms that in fact result from TB.

## What can we learn from interactions between COVID-19 and TB disease?

In a minority of patients, mainly elderly adults and/or those with underlying comorbidities, COVID-19 leads to severe pneumonitis and possibly long-term lung damage. We have much to learn about the long-term effect of this virus on lung function. The clinical presentation of lung disease in TB can be different, yet important interactions between the two diseases can be anticipated and need to be understood. To be diagnosed with COVID-19 does not exclude underlying TB, and in TB endemic settings particular attention should now be paid to this.

There are no data currently to inform us of the outcomes of co-infection, or that of COVID-19 in known TB patients. This information will emerge once the COVID-19 pandemic reaches the TB endemic areas of Africa and Asia. Data on long term sequelae for lung health in TB patients are sparse, and going forward, research studies (both laboratory and clinic-based) need to take COVID-19 into account as a possible co-factor in the interpretation of their results. Post-TB lung damage was an area that was until recently largely ignored. Hopefully this will now change given the potential for such patients to do worse with COVID-19 disease.

Similar considerations need to be given to COVID-19/HIV co-infections or underlying TB/HIV co-infection. There needs to be a particular emphasis on possible drug-drug interactions in individuals on medication for TB and/or HIV, who also may be using additional anti-virals and immune-modulating agents which show promise against COVID-19.

## What lessons for control can TB and COVID-19 learn from each other?

The impact of COVID-19 on populations and health systems in Africa will be very broad. The response, therefore, needs to be equally comprehensive and long term, rather than focused on just COVID-19. Countries in Africa will no doubt need help from the rich nations to mount a robust, sustained and regional response. Therefore, “it is time” for African countries to galvanize global attention and advocacy right now to substantially increase their investments toward the provision of universal health care, and to comprehensively strengthen their national health systems, with a particular focus on the primary health care and provision of essential diagnostics to the poorest and the least advantaged in society. This should be a wake-up call for countries in Africa to substantially increase their investments in health.

Tuberculosis control has only been possible through robust and consistent diagnosis, accompanied by contact tracing, including in households. This has also been implemented in the early stages of the COVID-19 pandemic in many—but by no means all—countries in order to minimise spread. The associated social distancing has helped to “flatten the curve” in China, South Korea and will hopefully be successful in all of the settings where it is currently followed. Much depends on it right now. The COVID-19 pandemic reminds us of the importance of this approach to TB too—with prevention being better than cure.

Community engagement has proven essential in TB control to address stigma, which has already been associated with COVID-19. Mitigation strategies that proved to be successful in TB might also assist in the community control of COVID-19 although the infection dynamics are very different.

The incredibly rapid and fruitful interactions of the scientific community to address the COVID-19 epidemic show what can be done when an emergency arises and scientists are focused and work together, with serious research funds being provided over a short time frame. It is encouraging to see the many new initiatives that have already arisen from the COVID-19 pandemic, be it in modelling, artificial intelligence for clinical algorithms to predict disease severity, international clinical trial platforms, or drug and vaccine developments.

Neither COVID-19 nor TB respect geographical or scientific borders. Lessons learnt from this emergency need to be applied to other infectious diseases of global importance, including TB, where a similar emphasis on clinical and research activity would no doubt have a very large impact. Without it, we will never control TB. One positive from the COVID-19 pandemic may be that the world becomes more aware of the need to control and eliminate infections through sustained and joined-up working. Let’s keep the momentum going and not forget TB!

## Conclusions

Tuberculosis is just one of many areas in global public health that will be sidelined and adversely affected by the COVID-19 pandemic. It is important to start thinking critically about these effects, and develop comprehensive mitigation plans where possible. If not addressed, the 10 million incident TB cases and > 1 million deaths from TB that occur annually worldwide will increase, with the negative impact being worst in LMICs. A positive aspect to these two pandemics colliding is that people—communities, public health professionals and policy makers—can learn from each other. Perhaps we can look forward to a time when infectious diseases are taken even more seriously and the link between infectious disease and poverty is further recognised such that increased investment in their control results in societal structural changes that benefit all.

## Data Availability

Not applicable.

## Abbreviations

HICs: High income countries
IPT: Infection preventive therapy
LMICs: Low and middle income countries
TB: Tuberculosis

## Footnotes

1 This has been subsequently modelled by the Stop TB Partnership to suggest that the impact of 2 months of lockdown and 2 months recovery will add an excess 1.8 million cases of TB disease over the next 5 years http://www.stoptb.org/assets/documents/news/Modeling%20Report_1%20May%202020_FINAL.pdf.
